# Disparity in Birth Size of Ethiopian Preterm Infants in Comparison to International INTERGROWTH-21st Data

**DOI:** 10.1177/2333794X20973484

**Published:** 2020-11-20

**Authors:** Netsanet Workneh Gidi, Robert L. Goldenberg, Assaye K. Nigussie, Zelalem Tazu Bonger, Elizabeth M. McClure, Mahlet Abayneh, Matthias Siebeck, Orsolya Genzel-Boroviczény, Lulu M. Muhe

**Affiliations:** 1 Jimma University, Jimma, Ethiopia; 2 CIHLMU, Center for International Health, University Hospital, LMU Munich, Germany; 3 Columbia University, New York, NY, USA; 4 Bill and Melinda Gates Foundation, Seattle, WA, USA; 5 Addis Ababa University, Addis Ababa, Ethiopia; 6 RTI International, Durham, NC, USA; 7 St Paul’s Hospital Millennium Medical College, Addis Ababa, Ethiopia; 8 Medical Center of the University of Munich, Munich, Germany

**Keywords:** preterm infants, anthropometric measurements, size at birth, SGA

## Abstract

*Background*. Patterns of fetal growth are largely influenced by environmental, nutritional, and socioeconomic factors more than differences in populations. The aim of this study was to assess anthropometric measurements of Ethiopian preterm infants at birth and compare the results with the international INTERGROWTH-21st data. *Patients and methods.* We analyzed anthropometric data on live-born singleton preterm infants enrolled in a hospital-based multicenter study of illness in preterm infants (SIP). Eligible newborns with gestational age of 28-36 weeks were included. Gestational age (GA) and sex-specific mean and standard deviations (SD), 10th, 50th, 90th, centile values for birth weight, length and head circumference (HC) were calculated and compared with INTERGROWTH-21st data. *Result*. A total of 2763 preterm infants were included in the study, 54% were male. The prevalence of small for GA (SGA) (<10th percentile) and large for GA (LGA) (>90th percentile) were 10.8% and 9.9%, respectively. In all 3 parameters, the mean values of boys were higher than of girls. Birth weight centiles were comparable to international averages at lower GA, then after GA of 32 weeks the 10th, 50th, and 90th centile values were 100-500 g less than the international averages. The head circumference centiles were mostly comparable, and the 90th centile values were greater than the international averages across the GA and in both sexes. *Conclusion.* The infants’ birth weights were smaller at higher GA, which may indicate maternal undernutrition in the third trimester of pregnancy. Strengthening antenatal nutrition counseling and providing nutrition supplementation might improve the birth weight.

## Introduction

Intrauterine growth measured by size at birth is one of the important determinant factors for neonatal survival.^1^ Weight, length and head circumference are among the anthropometric parameters widely used to measure intrauterine growth.^2^ Length of gestation is one of multiple factors associated with the size at birth. Fetal growth depends on genotype, sex, maternal health status, obstetric conditions, environmental factors, and geographic location^3^; however, only <3.5% of skeletal growth is attributed to differences in populations.^4^ Newborns are classified based on their weight, gestational age (GA) and weight adjusted for GA, to identify high risk infants such as those small for gestational age (SGA), and large for gestational age (LGA), often in need of specific interventions.^5^

Normal birth weight according to WHO, is from 2500 to 4000 g, while low birth weight (LBW) is defined as weight at birth <2500 g, very LBW (<1500 g) and extremely LBW (<1000 g).^6^ LBW contributes to most of neonatal and post neonatal mortality. Beyond infancy, survivors are at increased risk of growth failure and developmental problems at younger ages, and hypertension, type 2 diabetes, and heart disease during adulthood.^7^ Likewise, several studies from Ethiopia have reported a range of 9.1% to 28.3% prevalence of LBW in different parts of the country.^8-11^

Nutritional deficiency could be experienced in utero due to poor maternal diet that influences fetal nutrition, which results in smaller birth size and other detrimental birth outcomes and long term complications.^12^ The first 1000 days of life is a critical period, which determines the growth, immunologic, metabolic and neurodevelopmental outcome of individuals in subsequent years.^13,14^ The rate of undernutrition in under 5 children in Ethiopia is one of the highest in the world.^15^ Ephrem et al reported larger HC averages of Ethiopian children from birth to 24 months of age compared to WHO standards and recommended a different national reference range as the first choice of screening for hydrocephalus in Ethiopia.^16^ Data on the anthropometric parameters of preterm infants in most low-income countries are scarce, where the risk of neonatal death is very high.^17,18^

There is a large gap between birth outcomes of low income countries and high income countries.^7^ Birth size is largely determined by the maternal health, maternal nutrition and socioeconomic conditions than genetic differences.^4^ Comparison of the anthropometric values of the preterm infants with international data will reveal the status of intrauterine growth of Ethiopian preterm infants.

The aim of this study was to assess anthropometric measurements of Ethiopian preterm infants at birth and compare the results with the international INTERGROWTH-21st data.

## Methods

### Study Setting and Design

This was a hospital-based multi-center descriptive clinical study; using data from a study on causes of illness and death of preterm infants in Ethiopia (SIP). This study was undertaken in 5 government university hospitals in Ethiopia; Gondar University hospital (north Ethiopia), Jimma University Medical Center (south-west Ethiopia); and 3 hospitals in Addis Ababa, Black Lion Hospital, Saint Paul Millennium College Hospital, and Ghandi Memorial Hospital. The methodology paper of the protocol and the paper on major causes of death of preterm infants in Ethiopia have been published previously.^19,20^

### Study Participants

Live-born, singleton preterm newborns, <7 days of age born during the study period between 2016 and 2018, and whose parents gave informed consent, and were enrolled in the primary study. Both the healthy preterm infants and those who were admitted to neonatal intensive care units (NICUs) were included for analysis. For the purpose of this analysis, newborns who were older than 48 hours at time of enrollment and those with major/gross congenital anomalies and dysmorphic features were excluded as these conditions may affect anthropometric measurements. We have also excluded those infants with GA of <28 weeks as they were few in numbers.

### Study Procedures

The assessment of GA was done based on 3 methods (last menstrual period (LMP), physical examination using the Ballard Score, and ultrasound). When the difference between GA assessed by Ballard and that calculated from an accurate LMP was not greater than 2 weeks, the LMP GA was assumed to be correct. GA assessment was performed by trained physicians and anthropometric measurements were done according to the standard procedures. Birth weight was measured with calibrated digital weighing scales to the nearest 10 g. Head circumference and length were also measured to the nearest 0.1 cm. Data was collected using study specific forms.

### Ethical Approvals

The study was conducted after ethical approval was obtained from Addis Ababa University College of health Sciences institutional review board (Ethics ID: AAUMF 03-008), and LMU Institutional Review Board (Ethics ID: 19-649).

### Data Analysis

Data analysis was done by R software. Percentiles of anthropometric measurements were calculated. Con-tinuous variables were presented as means and standard deviations. Results are presented as mean differences, standard deviations (SD), and as means with 95% confidence interval (CI). The prevalence of SGA (<10th percentile) and LGA (>90th percentile) in this study was calculated based on WHO standards.^21^

## Result

**Figure 1.**
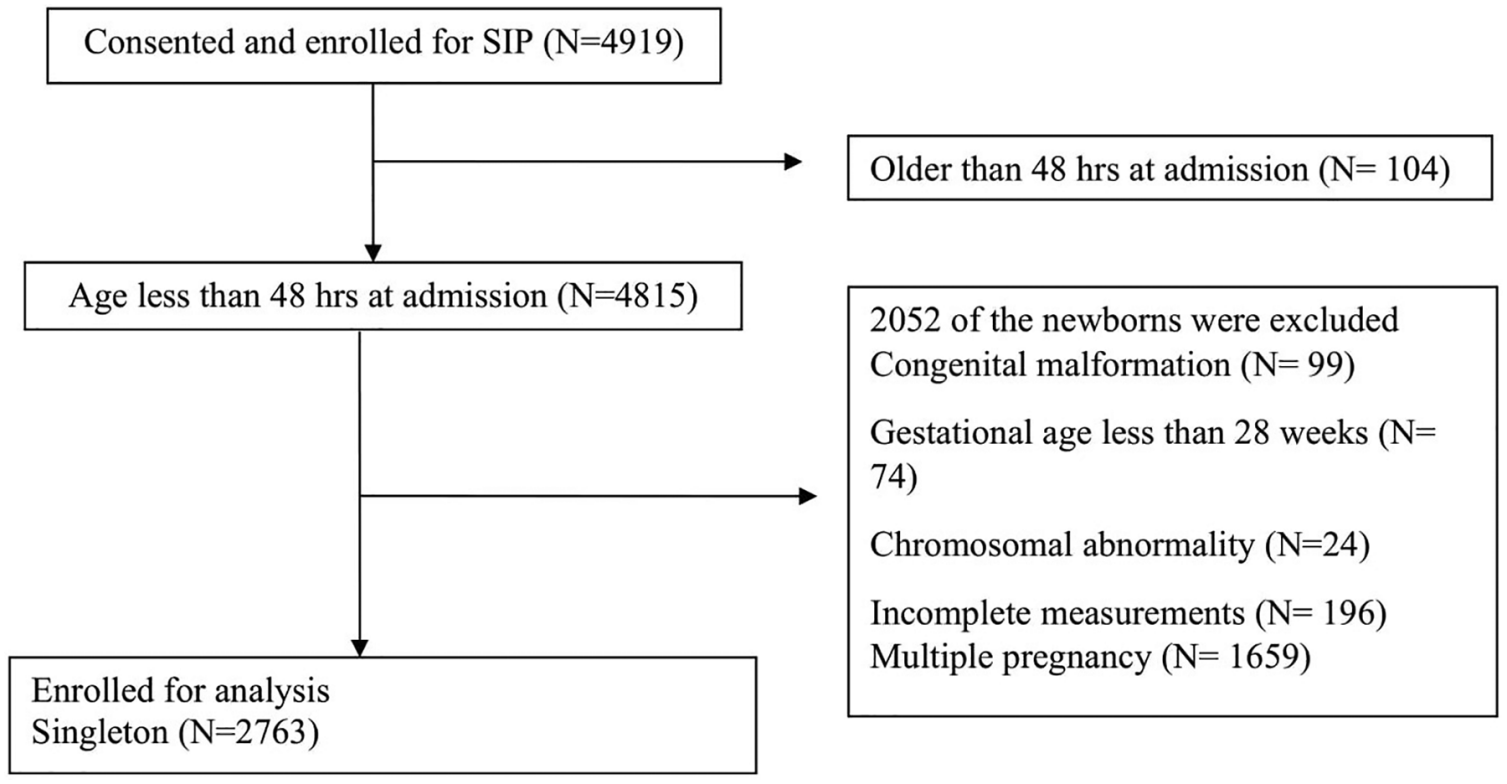
Flowchart of recruitment of study participants.

A total of 4919 were enrolled in the SIP study. Exclusions included those with major congenital anomalies (N = 24), those with GA <28 weeks (N = 74), those with incomplete anthropometric measurements (N = 196), postnatal age older than 48 hours at admission (N = 104) or a multiple pregnancy (N = 1659) (Figure 1). All 3 anthropometric measurements (birth weight, length and head circumference) were done at <48 hours of birth. From the total of 2763 preterm infants included in the study analyses, 54.2% were male. The majority of the infants were born in the 5 hospitals where the study was conducted, 58% of the infants were enrolled at Saint Paul and Black Lion hospitals; the rest were enrolled at Ghandi Memorial hospital, Gondar University Hospital and Jimma Medical Center.

The mean (SD) of the mothers’ age was 26 years (SD 5.4), and half of the mothers were younger than 25 years of age. More than one third 1048 (38%) were not able to read or write, while half had a formal education. Two fifths of the infants were late preterm 1082 (39%), while the rest were very preterm and moderate preterm, 896 (32%) and 785 (28%) respectively. More than one quarter 818 (32%) of the infants had a birth weight of <1500 g. The main reasons infants were brought to NICU were for prematurity, breathing problem and being cold to touch (Table 1).

**Table 1.** Maternal and Preterm Infants Characteristics.

Variables	Values
Maternal age (years), mean (SD)	26 (5.4)
Educational status No. (%)	
Not able to read and write	1048 (37.9)
Able to read and write	150 (5.4)
Formal education	1565 (56.6)
Sex No. (%)	
Male	1497 (54.2)
Female	1266 (45.8)
Gestational age (weeks), No. (%)	
28-31	785 (28.4)
32-33	896 (32.4)
34-36	1082 (39.2)
Birth weight (g), No. (%)	
≤1000	141 (5.1)
1001-1499	677 (24.5)
1500-2499	1715 (62.1)
≥2500	230 (8.3)
Common reasons for admission to NICU No. (%)*	
Prematurity	1949 (70.5)
Breathing problems	769 (27.8)
Cold to touch	758 (27.4)
Feeding difficulty	501 (18.1)
Referral from other facilities	331 (12.0)

Abbreviations: SD, standard deviation; NICU, neonatal intensive care unit.

* The percent does not add up to 100 since the infants had more one reason for admission.

GA and sex-specific means and standard deviations (SD) for birth weight, length and head circumference are shown in Table 2. In all 3 parameters, the mean values of boys were higher than of girls. The prevalence of SGA and LGA was comparable across the GAs, with overall prevalence of 10.8% and 9.9%, respectively (Table 3).

**Table 2.** Sex and Gestational Age-Specific Mean ± SD Values for Birth Weight, Length and Head Circumference, for Live Born Preterm Infants, Ethiopia, 2016-2018.

Gestational age in weeks (No.)	Boys			Girls		
	Birth weight (g) Mean ± SD	Length (cm) Mean ± SD	HC (cm) Mean ± SD	Birth weight (g) Mean ± SD	Length (cm) Mean ± SD	HC (cm) Mean ± SD
28 (64)	1172.6 (295.4)	37.7 (3.6)	27.8 (2.4)	1149.4 (328.0)	37.1 (3.8)	27.1 (2.0)
29 (134)	1246.0 (317.2)	38.3 (3.7)	28.1 (2.7)	1131.2 (260.7)	37 (3.7)	27.8 (2.8)
30 (118)	1386.7 (369.0)	39.7 (4.1)	29.0 (2.6)	1351.5 (344.8)	38.66 (4.4)	29.4 (3.5)
31 (248)	1524.7 (302.0)	40.3 (6.6)	29.7 (2.7)	1426.5 (300.0)	39.7 (3.3)	29.1 (2.7)
32 (221)	1628.6 (357.5)	41.8 (3.9)	30.9 (2.2)	1566.9 (345.7)	41.7 (3.8)	30.1 (2.1)
33 (427)	1744.7 (349.6)	42.4 (3.3)	30.9 (2.2)	1691.3 (360.0)	42.1 (3.5)	30.6 (2.1)
34 (469)	1902.7 (411.0)	43.2 (3.7)	31.9 (2.7)	1849.1 (395.4)	43.2 (3.6)	31.5 (2.9)
35 (646)	2096.1 (356.0)	44.4 (3.7)	32.5 (2.3)	2025.5 (364.0)	43.7 (3.9)	32.1 (2.4)
36 (436)	2205.7 (395.0)	45.1 (4.4)	33.0 (226.0)	2094.5 (390.0)	45 (4.0)	32.7 (2.7)

Abbreviations: SD, standard deviation; HC = head circumference.

**Table 3.** Prevalence of SGA and LGA Across the Categories of Live Born Preterm Infants, Ethiopia, 2016-2018.

Preterm category based on gestational age	Birth weight Mean (SD)	% of SGA (<10th centile) (95% CI)	% of LGA (>90th percentile) (95% CI)
Late preterm (34-36)	2102 (379)	10.2 (8.5,12.1)	9.3 (7.7, 11.2)
Moderate preterm (32-33)	1762 (391)	11.1 (9.4,13.1)	10.5 (8.8, 12.4)
Very preterm (28-31)	1351 (340)	11.2 (8.8,14.1)	9.8 (7.6, 12.5)
Total	1811 (467)	10.8 (9.7, 12)	9.9 (8.8, 11.1)

Abbreviations: SD, standard deviation; SGA, small for gestational age; LGA, large for gestational age.

The birth weights of the infants in this study were generally comparable with international standards at lower GA; then after GA of 32 weeks the 10th, 50th, and 90th centiles were 100-500 g less than the international averages (Tables 4 and 5). Whereas the HC values were mostly comparable to the values from Intergrowth-21, the 90^th^ centile values were found to be consistently higher than the international standards at all GAs in both sexes (Tables 6 and 7). The median and 90th centile of length at birth of the infants in this study were mostly comparable with international standards, and the 10th centile values were smaller at all GA in both sexes (Tables 8 and 9).

**Table 4.** Comparison of Ethiopian Singleton Preterm Infants’ Birth Weight in Kg (Boys) with International Data (INTERGROWTH-21st).

Gestational age	Ethiopian (SIP data)			International (INTERGROWTH-21st)		
	10th centile	50th centile	90th centile	10th centile	50th centile	90th centile
28	0.84	1.14	1.56	0.84	1.07	1.37
29	0.82	1.24	1.68	0.95	1.21	1.56
30	1.01	1.35	1.83	1.07	1.37	1.76
31	1.11	1.48	1.93	1.21	1.55	1.98
32	1.21	1.61	2.07	1.36	1.74	2.23
33	1.33	1.75	2.22	1.43	1.95	2.52
34	1.46	1.90	2.38	1.71	2.22	2.79
35	1.59	2.06	2.55	1.95	2.47	3.03
36	1.73	2.23	2.73	2.18	2.69	3.25

Abbreviation: SIP, study on causes of illness and death of preterm infants in Ethiopia.

**Table 5.** Comparison of Ethiopian Singleton Preterm Infants’ Birth Weight in Kg (Girls) with International Data (INTERGROWTH-21st).

Gestational age	Ethiopian (SIP data)			International (INTERGROWTH-21st)		
	10th centile	50th centile	90th centile	10th centile	50th centile	90th centile
28	0.76	1.05	1.45	0.79	1.01	1.30
29	0.84	1.16	1.57	0.90	1.15	1.47
30	0.94	1.27	1.70	1.01	1.30	1.66
31	1.04	1.40	1.84	1.14	1.46	1.87
32	1.15	1.54	1.99	1.28	1.64	2.11
33	1.28	1.69	2.15	1.41	1.86	2.35
34	1.40	1.84	2.32	1.68	2.13	2.64
35	1.53	1.99	2.47	1.92	2.38	2.89
36	1.64	2.12	2.59	2.14	2.60	3.12

Abbreviation: SIP, study on causes of illness and death of preterm infants in Ethiopia.

**Table 6.** Comparison of Ethiopian Singleton Preterm Infants’ Head Circumference in cm at Birth (Boys) with International Data (INTERGROWTH-21st).

Gestational age	Ethiopian (SIP data)			International (INTERGROWTH-21st)		
	10th centile	50th centile	90th centile	10th centile	50th centile	90th centile
28	24.6	27.5	30.6	23.9	25.9	27.9
29	25.4	28.2	31.3	24.8	26.8	28.8
30	26.8	28.9	31.9	25.7	27.7	29.7
31	27.0	29.6	32.5	26.6	28.6	30.6
32	27.8	30.3	33.1	27.4	29.4	31.4
33	28.6	31.0	33.7	29.1	30.9	32.7
34	29.3	31.7	34.3	29.8	31.5	33.2
35	30.0	32.3	34.9	30.4	32.0	33.7
36	30.7	33.0	35.4	30.9	32.5	34.2

Abbreviation: SIP, study on causes of illness and death of preterm infants in Ethiopia.

**Table 7.** Comparison of Ethiopian Singleton Preterm Infants’ Head Circumference in cm at birth (Girls) with International Data (INTERGROWTH-21st).

Gestational age	Ethiopian (SIP data)			International (INTERGROWTH-21st)		
	10th centile	50th centile	90th centile	10th centile	50th centile	90th centile
28	24.4	27.1	29.9	23.6	25.6	27.6
29	25.2	27.8	30.6	24.5	26.5	28.5
30	26.0	28.6	31.3	25.4	27.4	29.4
31	26.8	29.3	32.0	26.3	28.3	30.3
32	27.6	30.0	32.6	27.2	29.2	31.2
33	28.3	30.6	33.3	28.8	30.5	32.2
34	29.0	31.3	33.9	29.4	31.1	33.8
35	29.7	31.9	34.4	30.1	31.6	33.3
36	30.3	32.5	35.0	30.6	32.1	33.7

Abbreviation: SIP, Study on causes of illness and death of preterm infants in Ethiopia.

**Table 8.** Comparison of Ethiopian singleton preterm infants’ length in cm at birth (boys) with international data (INTERGROWTH-21st).

Gestational age	Ethiopian (SIP data)			International (INTERGROWTH-21st)		
	10th centile	50th centile	90th centile	10th centile	50th centile	90th centile
28	32.9	37.7	42.5	34.0	37.3	40.6
29	33.9	38.6	43.3	35.2	38.6	41.9
30	34.9	39.6	44.2	36.5	39.8	43.2
31	35.9	40.5	45.0	37.8	41.2	44.4
32	36.9	41.5	45.9	39.0	42.4	45.7
33	37.9	42.4	46.7	41.1	43.8	46.6
34	38.9	43.4	47.7	42.4	45.0	47.6
35	39.8	44.4	48.6	43.3	46.0	48.5
36	40.7	45.3	49.7	44.6	47.0	49.4

SIP= Study on causes of illness and death of preterm infants in Ethiopia.

**Table 9.** Comparison of Ethiopian Singleton Preterm Infants’ Length in cm at Birth (Girls) with International Data (INTERGROWTH-21st).

Gestational age	Ethiopian (SIP data)			International (INTERGROWTH-21st)		
	10th centile	50th centile	90th centile	10th centile	50th centile	90th centile
28	31.9	36.9	40.4	33.5	36.9	40.2
29	32.7	37.9	41.6	34.8	38.1	41.5
30	34.0	38.9	42.9	36.1	39.4	42.7
31	35.6	40.0	44.2	37.3	40.7	44.0
32	36.9	41.1	45.4	38.6	41.9	45.3
33	37.9	42.2	46.4	41.0	43.4	45.7
34	38.6	43.3	47.3	42.2	44.6	46.8
35	39.3	44.4	48.2	43.3	45.6	47.8
36	40.1	45.5	49.2	44.3	46.5	48.6

Abbreviation: SIP, study on causes of illness and death of preterm infants in Ethiopia.

## Discussion

Birth size has considerable significance in terms of determining the risk of death and extrauterine complications. In many low-income and middle-income countries, the proportion of neonates born SGA is generally higher than those who are born preterm; both conditions are associated with increased risk of death during the neonatal period and after.^7^ Survivors continue to be at risk of complications associated with nutrient deficiencies, common morbidities during infancy, developmental disorders and chronic diseases in adulthood.^7,22^

The means of all the 3 measurements were found to be higher in male infants than in female infants. This finding is similar to other reports.^23-25^ According to the findings of a multicenter international study “ INTERGROWTH-21st fetal growth standards”, variation in fetal growth across different populations is mostly dependent on environmental, nutritional, and socioeconomic factors; only <3.5% of the total variability of skeletal growth was due to differences between populations.^4^ Interpretation of anthropometric indices using local charts could lead to misclassification of infants and could negatively influence the diagnosis and treatment of the infants.^4^ In comparison with international data the birth weight centiles of the Ethiopian infants were similar at lower GA, but the infants born at later GA had smaller birth weights. Similar patterns of birth weight differences in centile values at later GA were reported by investigators from Indonesia and Australia.^24,25^ Fetal weight gain occurs fastest in the third trimester, factors affecting size at birth are observed to have a greater impact in later GA, and could explain the pattern of differences in birth weight of the infants across the GA compared to the international values.^26^ Inadequate nutritional intake of the mother at third trimester, and insufficient nutritional counseling of the mothers during antenatal care follow up could be the reason for the smaller size of the infants at later GAs.

Unlike birth weight, the differences in HC seem to be unaffected as the GA increases. The higher 90th centile values of HC observed in this study is similar to the findings of Ephrem et al^16^ They reported Ethiopian HC reference curves considerably higher than those of the WHO growth standards. This finding could be due to a genetic difference, or environmental factors influencing the HC, and may indicate the need for a population based chart for diagnosis of macrocephaly in Ethiopian preterm infants.

We used the definition of SGA suggested by a 1995 WHO expert committee as infants below the 10th centile of a birthweight-for-gestational-age, sex-specific reference population.^21^ In addition to complications of prematurity, preterm SGA infants are at increased risk of morbidity and mortality, 10-40 times greater risk of dying in the first month of life than term appropriate for GA (AGA) infants.^27^ In 2010 about 1.2% to 3.0% of the preterm births in low- and middle-income countries were estimated to be SGA, the SGA percentage in the current study is considerably higher (10.8%), however the this study was hospital based, and might not represent the prevalence of SGA in the general population.^28^

Limitations of this study include the fact that the data were hospital-based, rather than population-based, and infants <28 weeks of GA were excluded, because they were few in numbers. And the international growth standard (INTERGROWTH 21st) had exclusion criteria of mothers living at altitude greater than 1600 m. All the study sites in the current study have altitudes greater than 1600 m, which might have an influence on birth size. In addition, and INTERGROWTH data had fewer infants of GA <33.

## Conclusion

Birth weights of the infants in this study were comparable with the international averages at lower GA; however at later GA, the infants’ measurements were generally smaller. The finding that the infants were smaller at higher GA may indicate maternal undernutrition in the third trimester of pregnancy. Strengthening antenatal nutrition counseling and providing supplementation might increase the birth weight. Improving birth weight may have a positive impact on neonatal survival. Mostly, the lengths of the infants were smaller than international averages across the GAs; HC measurements were comparable, the 90th centile values were higher than international averages consistently in all GAs and in both sexes, which could indicate the need to develop a population-based HC charts for unbiased interpretation in clinical practice. The need for specific anthropometric standards for those who live at higher altitude might be a question worth investigating. The percentage of preterm SGA in this study was higher than the estimate in low- and middle-income countries, population based study is required to assess the burden of preterm SGA in this setting.
